# Low incidence of limb-girdle muscular dystrophy type 2C revealed by a mutation study in Japanese patients clinically diagnosed with DMD

**DOI:** 10.1186/1471-2350-11-49

**Published:** 2010-03-30

**Authors:** Yo Okizuka, Yasuhiro Takeshima, Kyoko Itoh, Zhujun Zhang, Hiroyuki Awano, Koichi Maruyama, Toshiyuki Kumagai, Mariko Yagi, Masafumi Matsuo

**Affiliations:** 1 Department of Pediatrics, Kobe University Graduate School of Medicine, Kobe 650-0017, Japan; 2 Department of Pathology and Applied Neurobiology, Graduate School of Medical Science, Kyoto Prefectural University of Medicine, Kyoto 602-8566, Japan; 3 Aichi Welfare Center for Persons with Developmental Disabilities, Aichi 480-0392, Japan

## Abstract

**Background:**

Limb-girdle muscular dystrophy type 2C (LGMD2C) is an autosomal recessive muscle dystrophy that resembles Duchenne muscular dystrophy (DMD). Although DMD is known to affect one in every 3500 males regardless of race, a widespread founder mutation causing LGMD2C has been described in North Africa. However, the incidence of LGMD2C in Japanese has been unknown because the genetic background remains uncharacterized in many patients clinically diagnosed with DMD.

**Methods:**

We enrolled 324 patients referred to the Kobe University Hospital with suspected DMD. Mutations in the dystrophin or the SGCG genes were analyzed using not only genomic DNA but also cDNA.

**Results:**

In 322 of the 324 patients, responsible mutations in the dystrophin were successfully revealed, confirming DMD diagnosis. The remaining two patients had normal dystrophin expression but absence of γ-sarcoglycan in skeletal muscle. Mutation analysis of the SGCG gene revealed homozygous deletion of exon 6 in one patient, while the other had a novel single nucleotide insertion in exon 7 in one allele and deletion of exon 6 in the other allele. These mutations created a stop codon that led to a γ-sarcoglycan deficiency, and we therefore diagnosed these two patients as having LGMD2C. Thus, the relative incidence of LGMD2C among Japanese DMD-like patients can be calculated as 1 in 161 patients suspected to have DMD (2 of 324 patients = 0.6%). Taking into consideration the DMD incidence for the overall population (1/3,500 males), the incidence of LGMD2C can be estimated as 1 per 560,000 or 1.8 per million.

**Conclusions:**

To the best of our knowledge, this is the first study to demonstrate a low incidence of LGMD2C in the Japanese population.

## Background

Duchenne muscular dystrophy (DMD; OMIM#310200) is the most common inherited muscular dystrophy, and affects 1 in every 3,500 males, regardless of race. DMD is caused by a mutation in the dystrophin gene on the short arm of the X chromosome and is characterized by the absence of dystrophin in skeletal muscle. Those affected by DMD develop muscle weakness by the age of 4 or 5, followed by progressive muscle wasting that ultimately leads to patients being wheelchair bound by the age of 12. In addition, calf hypertrophy and lumbar lordosis are also observed. DMD patients succumb to either cardiac or respiratory failure secondary to the disease during their twenties [1].

Limb-girdle muscular dystrophy type 2C (LGMD2C) (OMIM # 253700) is an autosomal recessive disorder caused by mutations in the SGCG gene, which encodes γ-sarcoglycan. It is characterized by a childhood onset of progressive muscular dystrophy. The mean age of onset is 5.3 years, and half of these patients lose ambulation by the age of 12. Calf hypertrophy and lumbar lordosis are common [2]. Based on these clinical findings, LGMD2C is referred to as a severe childhood autosomal recessive muscular dystrophy or as a Duchenne muscular dystrophy (DMD)-like autosomal recessive disease [3].

Unlike DMD, LGMD2C shows geographical difference in its incidence. The highest incidence of LGMD2C has been reported in North Africa as a result of a founder mutation in the SGCG gene [4]. Numerous studies have summarized the clinical and pathological features of LGMDs outside of North Africa. These studies have reported at least 19 subtypes, with 7 exhibiting autosomal dominant (LGMD1A to E) and 12 exhibiting autosomal recessive (LGMD2A to J) patterns of inheritance [5]. Previous studies have determined the prevalence of LGMD to range from 8.1 per million in a nationwide study in The Netherlands [6] to 40 per million in a worldwide survey [7]. However, the incidence of subtype LGMD2C has yet to be determined. In the Bulgarian Roma (Gypsy) population, one founder mutation has been reported to be common [8]. Other than in the geographical areas associated with founder mutations, only limited numbers of LGMD2C cases have been reported. For example, only nine and seven LGMD2C patients have been described among large numbers of patients examined in Italy [5] and the USA [9], respectively.

Since differentiation of LGMD2C from DMD has not been considered a major problem in current clinical practice, strong efforts to differentiate the two conditions have not been made. Dystrophin restoration therapy for DMD by either inducing exon skipping [10,11] or by suppressing nonsense mutations [12] appears to be close to clinical implementation. However, before there can be any clinical application of these technologies, it is essential that DMD be confirmed at the molecular level.

Kobe University Hospital contains a DMD clinic that examines patients suspected to have the disease from all over Japan, especially from the western part of the country. We herein report on two LDMD2C patients that were found among a group of 324 Japanese patients suspected to have DMD. We accordingly estimate the incidence of LGMD2C in the Japanese population to be 1 per 560,000.

## Methods

### Patients

Boys were enrolled in this cohort if they had been referred to the Kobe University Hospital with a tentative clinical DMD diagnosis based on strongly elevated levels of serum creatine kinase (CK) activity. Patients ranged in age from 0 - 7 years. We performed an extensive analysis on mutations in the dystrophin gene and were able to molecularly identify a mutation in the dystrophin gene in 322 patients with DMD-like disease (manuscript in preparation). However, in two other patients, no mutations were noted in the dystrophin gene. After obtaining informed consent from their parents, further examinations were conducted on these patients.

### Methods

#### Muscle biopsy

Muscle samples were obtained from the quadriceps of each patient. Standard histochemical stains including hematoxylin and eosin (H-E), Gomori trichrome, NADH tetrazolium reductase, succinate dehydrogenase, periodic acid-Schiff, acid phosphatase, adenosine triphosphatase at pH 4.3 and 9.4, cytochrome c oxidase, and alkaline phosphatase were conducted. Immunohistochemical stains for α- and β-dystroglycan; α-, β-, γ-, and δ-sarcoglycan; dystrophin; and merosin were performed using their respective monoclonal antibodies (Novocastra, Newcastle upon Tyne, United Kingdom, and Millipore, Billerica, MA, USA).

#### Gene analysis

Under an institutionally approved protocol, DNA was extracted from blood samples that were obtained from the probands and all available family members. For examination of the SGCG gene, eight sets of primers (Table 1) were designed to amplify eight exons, with the amplified PCR products then directly sequenced. Reverse transcription PCR (RT-PCR) was used to analyze the SGCG mRNA expressed in lymphocytes or skeletal muscle, as previously described [13]. Full-length SGCG cDNA was amplified as two separate, partially overlapping fragments by using two sets of primers (Table 1). The amplified products were then directly sequenced.

**Table 1:** Primer sequences

	forward primer	reverse primer
gSG 1F/R	atgcgaagagctgtgtcctg	tgccaccaaagaagaaagaa

gSG 2F/R	gcctccctcattccctctct	tcagagccagacagcaaagaa

gSG 3F/R	ggagaaatgcagaaaaggtggt	tgtgcacatgtatgcgcttt

gSG 4F/R	cagcacctattttgcaaattttataaatc	gcaccatgatgaagctggactc

gSG 5F/R	tagggttgacgtggcatgtg	tgtgtactccatggaatgttgtg

gSG 6F/R	gcctgctaatttgtaattgctttg	gcggaaagtcttgaaaataaagg

gSG 7F/R	ttttgtgcttcttttcctcatctc	cagtaggaggctgatctgtga

gSG 8F/R	ccttaactcttcgtctcccatctt	gcgtttacgtcccatccacgctgcc

gαDG 2F/R	tccaactcggggtagatgtttt	acttgaaaaggaaaagccacca

mSG 1F/7R	cattctgtctgtggtagagctcgg	gtttcagcatcaagcacaagcattcc

mSG 5F/8R	aaatggtagaagtccagaatcaaca	gcgtttacttcccatccacgctgc

#### Quantitative PCR

Genomic dosage of the exons of the SGCG gene was assessed by a semiquantitative multiplex PCR, as previously described [14]. Eight fragments encompassing exons 1 to 8 of the SGCG gene and one fragment encompassing exon 2 of the α-dystroglycan gene were co-amplified using two PCR reactions that employed six sets of primers (Table 1). PCR products were separated by capillary electrophoresis (Agilent 2001 Bioanalyzer with DNA 1000 Lab Chips, Agilent Technologies, Palo Alto, CA, USA). The amount of PCR product derived from the SGCG exons was quantified by measuring their peak areas followed by calculating the ratio of these areas to that found for the α-dystroglycan exon 2.

## Results

The two male patients with clinical diagnosis of DMD were incidentally found to have marked elevations of serum CK levels (more than 50 times higher than control) in early childhood, despite a negative family history for muscular dystrophy. To confirm the clinical diagnosis of DMD, dystrophin gene mutations were extensively searched for using not only genomic DNA but also mRNA. However, no mutations could be identified, even when we included a deep intron mutation [15].

**Figure 1:**
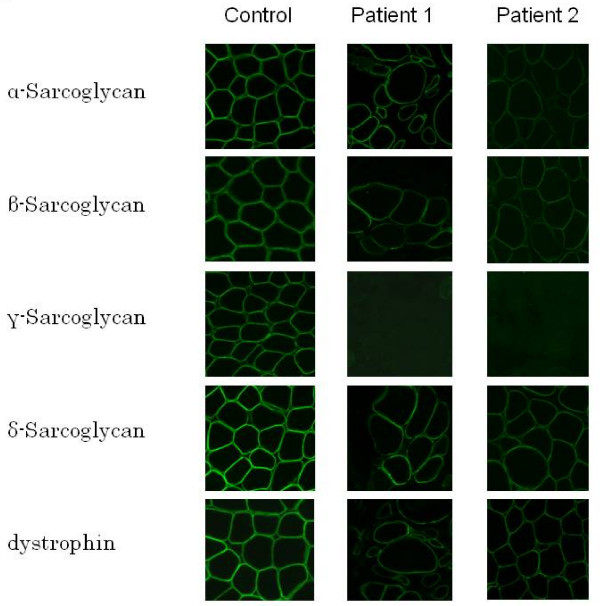
**Immunostaining of skeletal muscle**. Results of immunohistochemical examination using dystrophin, α-, β-, γ-, and δ-sarcoglycans and dystrophin antibodies are shown. There was clear staining of every protein along the plasma membrane in the control. In patient 1, γ-sarcoglycan was completely absent, and there was a mild reduction of α-sarcoglycan. Both β-sarcoglycan and δ-sarcoglycan were found to be almost entirely intact. There was clear staining of dystrophin in patient 1. In patient 2, γ-sarcoglycan was completely absent, and a patchy reduction in both α- and β-sarcoglycan was observed. Dystrophin, δ-sarcoglycan, and merosin staining patterns appeared normal.

**Figure 2:**
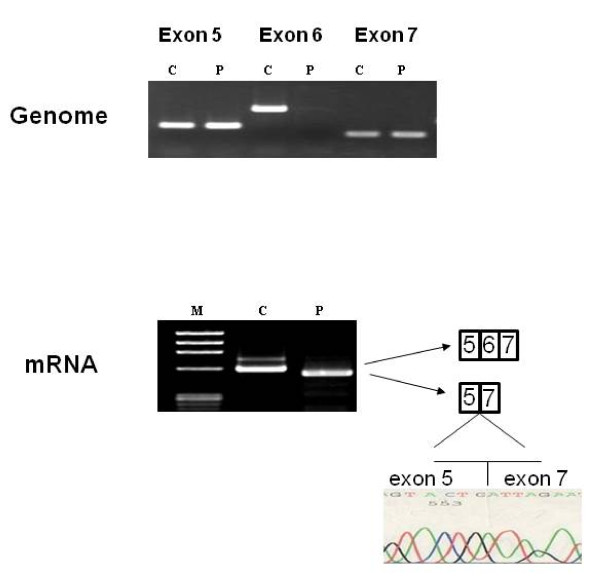
**Mutation analysis of the SGCG gene in patient 1**. Amplification products of exons 5, 6, and 7 are shown (c; control, p: patient) in the upper part of the figure. Although PCR amplification of eight exons of the SGCG gene was conducted, no products were obtained from the fragment encompassing exon 6 (Exon 6 p). For the other exons, the products of the SGCG gene met size expectations. Results of SGCG mRNA analysis are shown in the lower part of the figure. RT-PCR (c; control, p: patient) was used to analyze the muscle mRNA. The fragment encompassing exons 5 to 8 was amplified as a small-sized product in patient 1. Sequencing of the product showed a complete absence of exon 6. It was concluded that patient 1 had a homozygous deletion of exon 6 in the SGCG gene.

To clarify pathological changes in these two patients, muscle biopsies were performed. In patient 1 (KUCG 527), H-E staining revealed marked replacement of muscle by adipose tissue along with increases in endomysial connective tissue. We also found a few muscle fibers that were remarkably different in size (data not shown). In spite of the clinical diagnosis of DMD, the immunostaining pattern for both dystrophin and merosin staining was completely normal. Unexpectedly, there was no staining for γ-sarcoglycan, while there was only a mild reduction for α-sarcoglycan and almost normal results for β- and δ-sarcoglycan (Figure 1). In order to confirm the γ-sarcoglycan deficiency, we looked for mutations in the SGCG gene. When PCR was used to amplify the eight exons that encompassed the regions of the SGCG gene, all regions other than exon 6 could be obtained. This suggests a homozygous deletion of exon 6 (Figure 2). To confirm this, we used RT-PCR to analyze the SGCG mRNA from the patient's muscle. Amplification of the fragment encompassing exons 5 to 8 resulted in a small-sized product. Subsequent sequencing of this product revealed a complete absence of the exon 6 sequence (data not shown). Therefore, we concluded that this patient had a homozygous deletion of exon 6 in the SGCG gene. In addition, both the patient's mother and father were found to carry this deletion in one allele (data not shown). Because the exon 6 deletion removes 73 bp (nt 506 - 578) from the mRNA, it was expected that a stop codon would appear in exon 7, thereby leading to γ-sarcoglycan deficiency. It became clear that this patient had LGMD2C rather than DMD.

**Figure 3:**
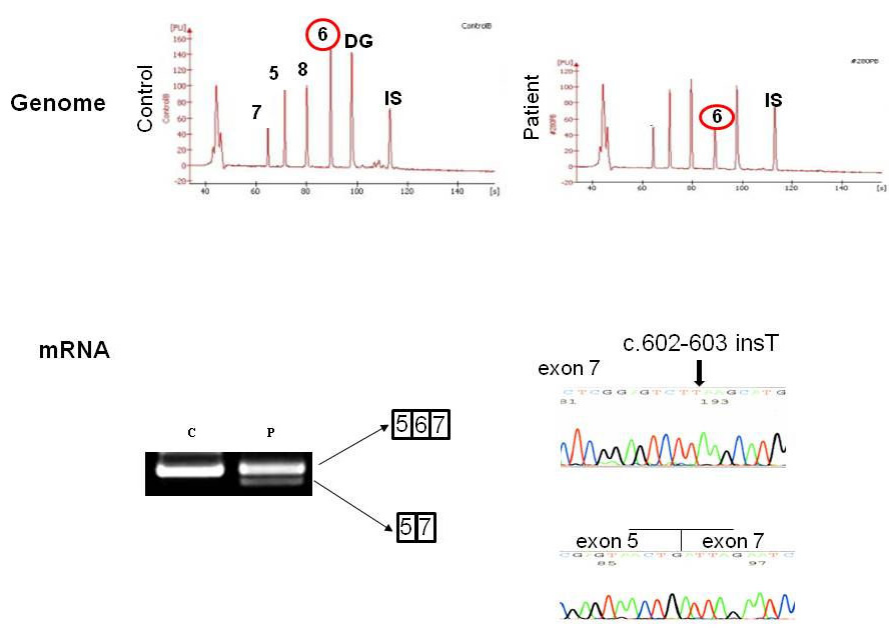
**Mutation analysis of the SGCG gene in patient 2**. Capillary electrophoretic patterns of the PCR products are shown in the upper portion of the figure. Five genomic regions were co-amplified in one PCR reaction, with the products separated using capillary electrophoresis. The position of each of the amplified products of exons 5, 6, 7, and 8 of the SGCG gene and exon 2 of the α-dystroglycan gene (DG) are marked. In patient 2, the peak area of exon 6 is nearly half that of the control, indicating a heterozygous deletion of exon 6. IS refers to a 1,500-bp marker. The amplified fragments that encompassed exons 5 to 8 of the SGCG mRNA are shown in the lower part of the figure (c; control, p; patient). Two different product sizes were obtained from the patient. Sequencing of the products disclosed that one exhibited a complete absence of exon 6, while the other included a nucleotide insertion in exon 7 (c.602_603insT). Thus, in this patient there was both a heterozygous nucleotide insertion in exon 7 and a heterozygous deletion of exon 6 of the SGCG gene.

In patient 2 (KUCG 280), H-E staining indicated excess variability of muscle fiber size, clusters of regenerating fibers, degenerating fibers, some acutely necrotic fibers, and scattered inflammatory cells. Immunohistochemistry revealed the absence of γ-sarcoglycan and a patchy reduction in α- and β-sarcoglycan. The staining patterns for dystrophin, δ-sarcoglycan, and merosin all appeared normal (Figure 1). Since the findings suggested γ-sarcoglycan deficiency, PCR amplification of the patient's genomic DNA was performed for all eight exons in the SGCG gene. Amplification of all fragments resulted in products that were of the expected size, and thus, subsequently could be used for direct sequencing. With the exception of exon 7, sequencing of the amplified products demonstrated a completely normal sequence. In exon 7, subcloning of an ambiguous sequence of the amplified product resulted in one clone with a completely normal sequence and a second clone that contained a novel single T nucleotide insertion between nt 602 and 603 within exon 7 (c.602_603insT). This mutation created a stop codon in exon 7. The patient's mother was heterozygous for this mutation, whereas the father had a normal exon 7 sequence (data not shown). Since patient 1 had a homologous deletion of exon 6 of the SGCG gene, we supposed that patient 2 carried this deletion in one allele. When semi-quantitative PCR amplification of exon 6 of the patient's SGCG gene was performed, the results showed nearly half the genomic dose for the exon 6 encompassing region, indicating a heterozygous deletion of exon 6 (Figure 3). Further analysis of the SGCG mRNA from the patient's muscle revealed two kinds of mRNA: one exhibited an exon 6 deletion and the other demonstrated the above-mentioned novel single nucleotide insertion within exon 7 (Figure 3). Therefore, the γ-sarcoglycan deficiency in this patient was caused by a hemizygous c.602-603insT in exon 7 along with the deletion of exon 6 on the other allele.

To date, our analysis of patients suspected to have DMD has revealed that two of the entire cohort examined can be regarded as having LGMD2C. Thus, the relative incidence of LGMD2C among Japanese patients suspected to have DMD can be calculated as 1 in 161 (2 of 324 patients = 0.6%). When the DMD incidence is taken into consideration for the overall population (1/3,500 males), the incidence of LGMD2C can be estimated as 1 per 560,000 or 1.8 per million.

## Discussion

This is the first comprehensive study that has been able to definitively clarify the incidence of LGMD2C among patients suspected to have DMD. In our cohort of 324 Japanese children clinically diagnosed with DMD, two were diagnosed as having LGMD2C. The incidence of LGMD2C among this cohort was calculated as 1 in 161 (0.6%) and the incidence in the Japanese population was estimated at 1 per 0.56 million people. The relative proportions of all LGMDs, including LGMD2C, have been previously reported for various regions of the world [5,9]. However, these reports did not describe the relative prevalence of LGMD2C to DMD. The present study is the first to describe the incidence of LGMG2C in one race. The incidence of severe LGMDs was estimated to be 11.8% in a German study of patients with severe muscular dystrophy with early childhood onset [16] and about 8 to 12% in a Brazilian study of males with a clinical diagnosis of DMD [17]. In comparison, our results indicate a much lower incidence (0.6%) of LGMD2C among patients suspected to have DMD. However, the low prevalence of LGMD2C in Japanese is in accordance with the few reports that have examined LCMD2C patients from Japan [18,19]. Regardless of these differences, the potential presence of LGMD2C needs to be considered when making a differential diagnosis of DMD, even in Japan.

Although originally reported to be a severe autosomal recessive muscular dystrophy that resembles DMD, LGMD2C has since been reported to have a heterogenous clinical course, even with identical mutations [19]. Initially, both of our LGMD2C patients were tentatively diagnosed as having DMD, even though both patients showed only very mild muscle weakness during the original observation period. A clinical hallmark for differentiating DMD and LGMD2C involves the inheritance pattern. However, this is impossible to determine in sporadic cases in males. In the current cases, the failure to determine any dystrophin gene mutations that were responsible for DMD led to immunohistochemical examination of muscle tissue. Quite unexpectedly, we found normal staining for dystrophin in these patients (Figure 1). This finding proved to be the key for diagnosing the complete or near-complete absence of γ-sarcoglycan deficiency, which led to our using genetic analysis to definitively prove the γ-sarcoglycan deficiency.

It has been reported that residual sarcoglycan expression is highly variable, and that this makes it difficult to accurately predict the genotype [2,20]. In addition, both sarcoglycanopathy and DMD were reported to show weak staining of all types of sarcoglycan complexes [20]. On the other hand, patients with LGMD2C have been reported to show a significant reduction or a complete absence of γ-sarcoglycan staining in conjunction with reduced or only partially preserved staining of the other sarcoglycan proteins [2,5,9,21,22]. In the present two cases, we observed a marked reduction of γ-sarcoglycan, in addition to finding a reduction of staining intensity for other members of the sarcoglycan complex (Figure 1).

Since the maintenance of the carboxyl terminus of γ-sarcoglycan is important for both the processing and stability of the protein, mutations in the extracellular domain of γ-sarcoglycan can lead to an absence of protein expression [4]. While the most common cause of LGMD2C is thought to be a homozygous del521T in exon 6 of the SGCG gene, this mutation has not been reported in Japanese. However, in the current study we identified a novel c.602_603insT mutation. We identified a homozygous deletion of exon 6 of the SGCG gene in patient 1. We also identified the exon 6 deletion in three of four alleles in our two Japanese LGMD2C patients. This mutation has been previously reported to occur as a hemizygous condition in Japanese [19]. In Europe, this exon 6 deletion has been reported in both homozygous and hemizygous conditions [23]. Taken together, these previous findings suggest that exon 6 can be considered prone to deletions. Although our identification of the exon 6 deletion in one allele was initially difficult, we have now been able to successfully detect deletions in one allele by using semiquantitative PCR amplification (Figure 3). Therefore, use of this methodology may help to increase the mutation detection rate in patients suspected to have DMD, and in addition, help to correctly identify LGMD2C. Being able to successfully identify LGMD2C patients in the future will help to ensure correct DMD diagnosis and proper implementation of therapy in patients who do indeed have DMD.

## Conclusion

This is the first comprehensive study to describe the prevalence of LGMD2C in one race from mutation study results on patients suspected to have DMD. The incidence of LGMD2C in the Japanese population was estimated to be 1 per 560,000.

## Competing interests

The authors declare that they have no competing interests.

## Authors' contributions

YO performed the molecular genetic studies, participated in the sequence alignment and drafted the manuscript. KI carried out the immunoassays. ZZ and HA participated in the sequence alignment. YT, MY, KM and TK participated in the design of the study. MM conceived of the study, and participated in its design and coordination and helped to draft the manuscript. All authors read and approved the final manuscript.

## Pre-publication history

The pre-publication history for this paper can be accessed here:

http://www.biomedcentral.com/1471-2350/11/49/prepub
